# Tissue nonspecific and intestinal alkaline phosphatase crosstalk: a missing link in hypophosphatasia pathophysiology?

**DOI:** 10.1186/s12967-026-07791-1

**Published:** 2026-02-08

**Authors:** Luis Martínez-Heredia, Trinidad González-Cejudo, María Carmen Andreo-López, Victoria Contreras-Bolívar, Cristina García-Fontana, Beatriz García-Fontana, Manuel Muñoz-Torres

**Affiliations:** 1 Instituto de Investigación Biosanitaria de Granada (Ibs. Granada), Granada, 18012 Spain; 2 CIBER on Frailty and Healthy Aging (CIBERFES), Instituto de Salud Carlos III, Madrid, 18012 Spain; 3 Clinical Analysis Unit, University Hospital Clínico San Cecilio, Granada, 18016 Spain; 4 Endocrinology and Nutrition Unit, University Hospital Clínico San Cecilio, Granada, 18016 Spain; 5 Department of Medicine, University of Granada, Granada, 18016 Spain

**Keywords:** TNSALP, IAP, Hypophosphatasia, Inflammation, Gut, Serum, Stool

## Abstract

**Background:**

Tissue-nonspecific alkaline phosphatase (TNSALP) and intestinal alkaline phosphatase (IAP) are functionally similar enzymes, but their relationship in hypophosphatasia (HPP) remains unexplored. This study investigated the impact of HPP—a condition caused by ALPL gene mutations that impair TNSALP function—on serum and fecal IAP activity.

**Methods:**

Total alkaline phosphatase (ALP) activity and isoenzyme-specific activities (using selective inhibitors: L-homoarginine for TNSALP, L-phenylalanine for IAP) were measured in serum and stool samples from 30 HPP patients and 30 matched healthy controls, alongside biochemical parameters correlations.

**Results:**

In serum, IAP activity showed a non-significant decrease in HPP patients compared to controls, while TNSALP and total ALP activity were reduced in HPP patients. In stools, both total ALP and IAP activities were significantly decreased compared to the control group. Multivariate linear regression revealed a strong positive association between TNSALP and IAP in both serum and feces, independent of age and sex. In serum, TNSALP and IAP were key predictors of total ALP activity (B = 0.876 and B = 0.745, respectively; *p* < 0.001; R² = 0.9396), with TNSALP also predicting serum IAP levels (B = 0.164; *p* < 0.001). In feces, IAP was the strongest predictor of total ALP activity (B = 0.921; *p* < 0.001), and fecal TNSALP strongly predicted IAP levels (B = 0.883; *p* < 0.001). Serum TNSALP activity correlated with bone metabolism markers, inflammation, underscoring its potential systemic role.

**Conclusions:**

IAP does not seem to compensate for reduced TNSALP activity in HPP. Instead, their tight association suggests a coordinated regulation between the two isoenzymes, with diminished fecal IAP potentially contributing to gut inflammation in HPP. These findings clarify the interplay between TNSALP and IAP and their clinical implications.

**Supplementary Information:**

The online version contains supplementary material available at 10.1186/s12967-026-07791-1.

## Background

Tissue nonspecific alkaline phosphatase (TNSALP) is a homodimeric ectoenzyme belonging to the alkaline phosphatase family (EC 3.1.3.1) encoded by the *ALPL* gene [1]. In addition to TNSALP, the alkaline phosphatase family includes three tissue-specific isoenzymes: intestinal (IAP), placental (PALP), and germ-cell alkaline phosphatase (GCALP). These enzymes are encoded by the *ALPI*, *ALPP*, and *ALPP2* genes, respectively. This family of enzymes hydrolyzes monoester bonds to produce inorganic phosphate (Pi) [2]. TNSALP is a ubiquitous protein that is expressed mainly in bone, liver and kidney, although it is also expressed by other cell types, such as neutrophils [3], lymphocytes [4] and macrophages [5]. The enzyme hydrolyzes inorganic pyrophosphate to produce Pi for hydroxyapatite synthesis. It also dephosphorylates PLP, allowing its transport across the blood–brain barrier and its role in neurotransmitter production [6]. Moreover, TNSALP shares other substrates, such as bacterial lipopolysaccharides (LPS) or adenosine triphosphate (ATP), along with its intestinal isoenzyme (IAP) [7].

IAPs are highly expressed in the luminal vesicles of duodenal enterocytes and, to a lesser extent, in the jejunum, ileum, and colon. They contribute to intestinal barrier homeostasis by regulating lipid absorption, modulating bicarbonate secretion through ATP dephosphorylation, and detoxifying LPS [8, 9].

Mutations in the TNSALP gene results in a rare disease called hypophosphatasia (HPP) (OMIM 241510, 241500, 146300) [10]. The mild form has a prevalence of 1/2,430 in Europe [11], with a higher estimated prevalence of 1/1,692 in Spain [12]. The main symptoms of this disease involve impaired bone and dental mineralization. However, its clinical presentation is highly variable, ranging from completely asymptomatic cases to profound lack of mineralization and even death [13] Many features overlap with more common disorders, contributing to frequent underdiagnosis. The most common symptoms include premature tooth loss and fractures resulting from impaired mineralization [14, 15]However, muscle [16], renal [17], neuronal [18] and gastrointestinal complications are also frequently reported in HPP patients [12, 19].

On the other hand, loss-of-function mutations in the ALPI gene reduce IAP activity, compromising intestinal barrier integrity and increasing susceptibility to inflammatory bowel diseases such as ulcerative colitis. This reduction in IAP activity is also associated with compensatory upregulation of TNSALP expression [20]. However, it is still unknown whether IAP itself provides any compensatory effect in individuals carrying loss-of-function mutations in ALPL. This study therefore aims to investigate potential compensatory mechanisms involving IAP activity under conditions of absent or reduced TNSALP function.

## Materials and methods

### Study design

**Fig. 1:**
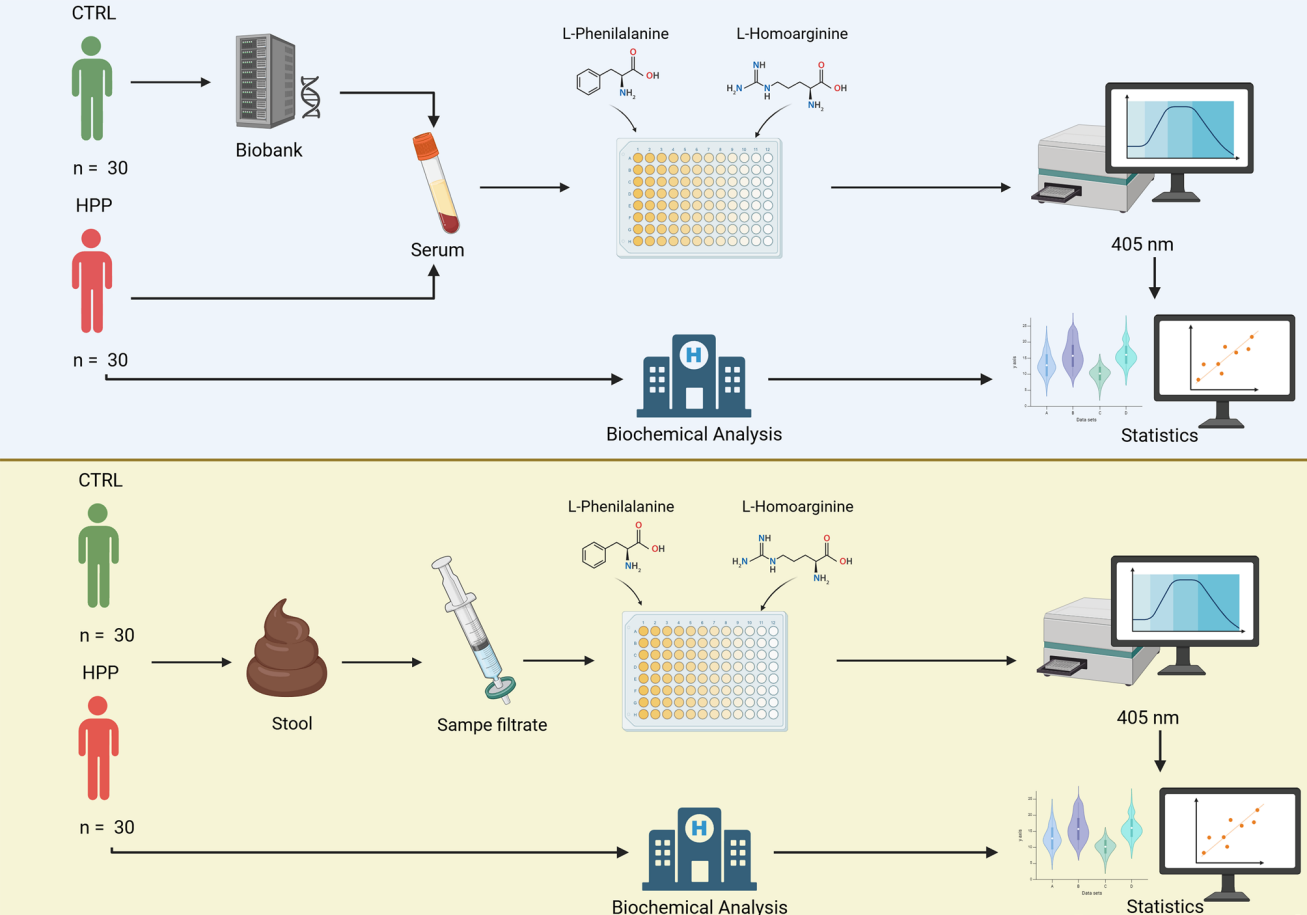
Graphical abstract

A cross-sectional study was conducted including 30 patients with hypophosphatasia (HPP) and 30 biobank donors for the analysis of serum alkaline phosphatase activity, as well as 30 HPP patients and 30 healthy donors for fecal activity determination, all matched by age and sex. Total alkaline phosphatase activity and the activity of the different isoenzymes were measured using a colorimetric kit. In addition, biochemical parameters were collected from HPP patients. Subsequently, an exploratory statistical analysis was performed, including comparisons of medians and linear regression analyses between patients and controls. Correlation analyses were conducted exclusively in the patient group and were limited to biochemical parameters, to evaluate the behavior of enzymatic activities. The study design is summarized in Fig. 1.

### Study population

Thirty HPP patients were recruited from the Endocrinology Unit at University Hospital Clínico San Cecilio of Granada from the 1st of January 2021 to the 31st of December 2024. HPP patients were diagnosed following the algorithm developed by Garcia-Fontana et al. [19]. and any of the participants presented with thyroid disease, disturbances in glucose homeostasis, including diabetes mellitus, or evidence of nutritional deficiencies. Serum and stool samples from HPP patients were collected and managed through a private collection registered under code C0007146 (https://biobancos.isciii.es/ListadoColecciones.aspx). Serum samples from age- and sex-matched healthy controls were provided by the Biobank and stool samples from healthy controls matched for age, and sex were used. All participants were informed and signed the corresponding informed consent, and the present study was approved by the Provincial Ethics Committee of Granada (CEI-Granada) on 01/01/2024 (ref: 1872-N-23), which respected the Data Protection Act and international regulations for human research (Helsinki Declaration, Council of Europe Convention on Human Rights and Biomedicine), as well as Spanish legislation (Law 14/2007 on Biomedical Research and other relevant national laws).

### Biochemical parameters

For biochemical measurements, venous blood samples and serum samples were collected in the morning after fasting overnight; these samples were obtained from each patient as part of routine clinical analyses and were measured via standard automated laboratory techniques. The measured inflammatory markers included calprotectin, C-reactive protein (CRP), interleukin 6 (IL6). For bone metabolism, the levels of bone-specific alkaline phosphatase (BALP), osteocalcin, N-terminal propeptide of type I collagen (P1NP), collagen type I C-terminal telopeptide (CTX), intact parathyroid hormone (iPTH), and 25-hydroxyvitamin D (25(OH)D) were determined. Plasma PLP levels were measured via high-pressure liquid chromatography (HPLC) at the Clinical Unit of the University Hospital Niño Jesús (Madrid).

### Alkaline phosphatase activity

Alkaline phosphatase (ALP) activity was measured in the serum and stool samples at 405 nm via an alkaline phosphatase detection kit (Abnova) according to the manufacturer’s recommended protocol. To determine the specific activities of the IAP and TNSALP isoenzymes, 10 mM specific inhibitors (L-phenylalanine and L-homoarginine, respectively) were added, and the samples were incubated at 37 °C for 15 min before their activities in the serum and stool samples were determined.

Serum samples were measured directly, and activities are expressed as IU/L. In contrast, stool samples were mechanically disrupted in a PBS solution containing protease inhibitors. The samples were then filtered through a 22 μm pore diameter filter to remove solid debris. Finally, the protein concentration was determined for each sample via the Bradford method, and the enzyme activity was expressed as IU/g.

### Statistical analysis

The statistical power calculation was performed via G*Power 3.1.9 software. A one-tailed post hoc analysis was conducted via Fisher’s exact test, assuming a significance level of 5% and observed group proportions of 70% and 30%, with 30 participants in each group; the statistical power reached 90%. As this was an exploratory study, non-parametric methods were used for hypothesis testing in order to ensure robustness against potential deviations from normality and small sample sizes. The detection of statistically significant differences using non-parametric tests suggests that the observed effects are robust. Comparisons between two groups were performed using the Wilcoxon test, while comparisons among more than two groups were carried out using the Kruskal-Wallis’ test. Multiple comparisons were adjusted using the false discovery rate (FDR) correction. Homoscedasticity and normality of residuals were assessed by visual inspection of residuals versus fitted values plots and Q–Q plots, respectively, in the multivariable linear regression models. Fecal data were log10-transformed to improve homoscedasticity. Variance Inflation Factor (VIF) values were calculated to find multicollinearity between variables. The model fit was measured by R^2^ and only those variables with a *p*-value < 0.05 and 95% Confidence Intervals (95% CI) were considered statistically significant. Spearman correlations were used to determine the correlations of each isoenzyme with the other biochemical variables of the HPP patients. P values less than 0.05 were considered significant. The figures and the statistical analysis were performed by RStudio.

## Results

### Total ALP, TNSALP and IAP activities in serum and stool samples

**Fig. 2:**
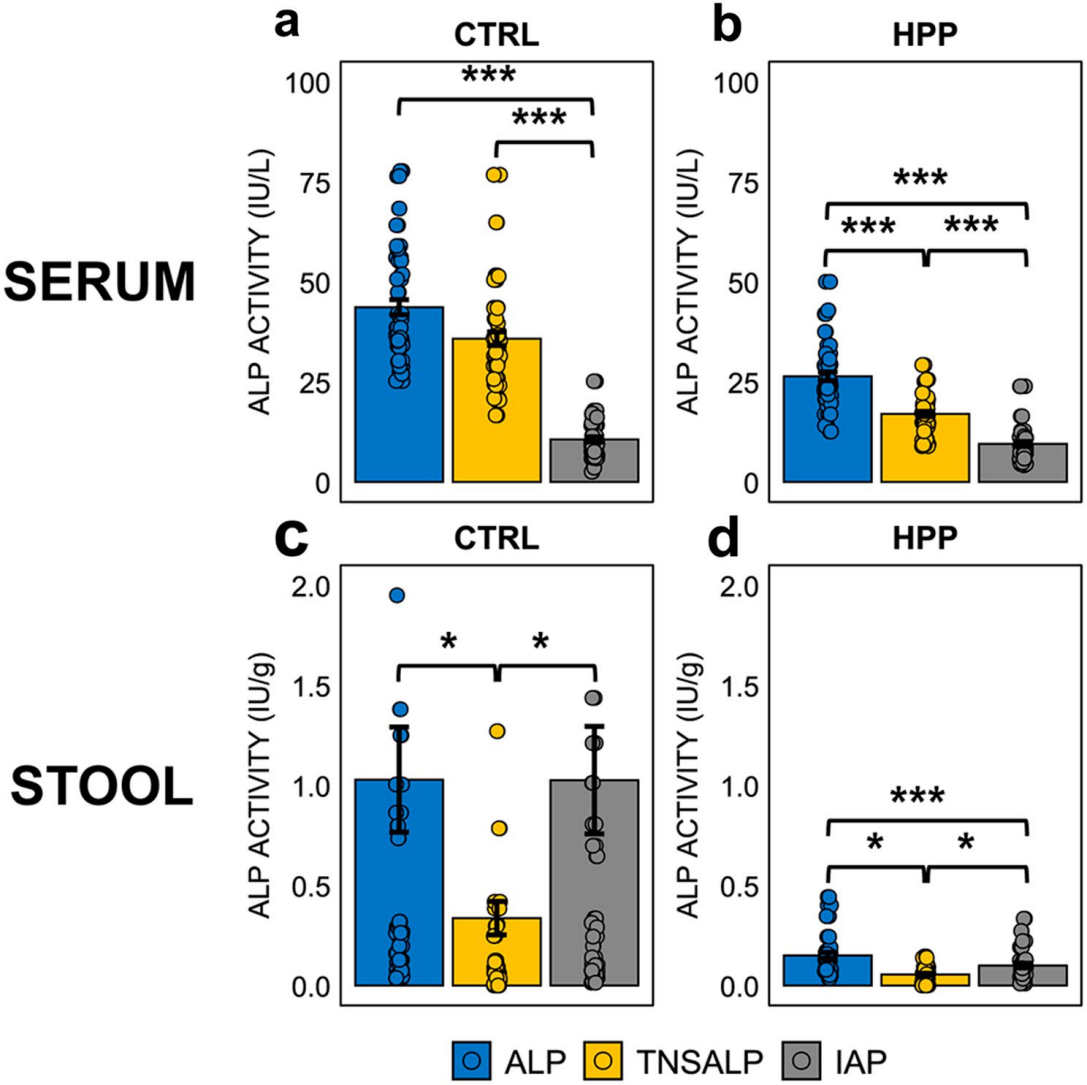
Total, tissue non-specific and intestinal ALP activities in serum from (**a**) healthy controls (*n* = 30) and (**b**) HPP patients (*n* = 30). Total, tissue non-specific and intestinal ALP activities in stool from (**c**) healthy donors (*n* = 29) and (**d**) HPP patients (*n* = 25). A Kruskal-Wallis test was performed for multiple comparisons and *p*-values were adjusted by FDR correction < 0.05 (*), < 0.01 (**), < 0.001 (***)

In the control group, the total serum ALP activity was 43.7 ± 2.69 IU/L with no significant differences from TNSALP activity (35.8 ± 2.4 IU/L; *p* = 0.138), which represents the main contribution to total ALP activity in serum. Regarding IAP activity, it was significantly lower (10.6 ± 0.89 IU/L) compared to the other two activities (*p* < 0.001 for both) (Fig. 2a).

In HPP patients, total ALP activity was 26.4 ± 1.56 IU/L, TNSALP activity was 17.0 ± 0.9 IU/L and IAP activity remained the lowest compared to other two activities (9.49 ± 0.9 IU/L). All comparisons were statistically significant (*p* < 0.001). Despite the presence of loss-of-function mutations, TNSALP activity persisted as the predominant enzymatic activity in serum, remaining higher than IAP activity in HPP patients (Fig. 2b).

In the control stool samples, total ALP activity was 1.03 ± 0.375 IU/g being the IAP activity the main contributor (1.03 ± 0.383 IU/g), whereas TNSALP activity was markedly lower compared to other activities (0.336 ± 0.12 IU/g; *p* = 0.031 vs. ALP, *p* = 0.044 vs. IAP) (Fig. 2c).

In the HPP group, total fecal ALP activity was 0.151 ± 0.021 IU/g. IAP activity was significantly lower compared to total ALP activity (0.099 ± 0.0157 IU/g; *p* = 0.0312) and TNSALP remained as the lowest activity (0.055 ± 0.008 IU/g) compared with total ALP activity (*p* < 0.001) and IAP (*p* = 0.045) (Fig. 2d).

Then, comparisons between controls and HPP patients regarding total ALP, TNSALP and IAP activities in both serum and stool were performed.

**Fig. 3:**
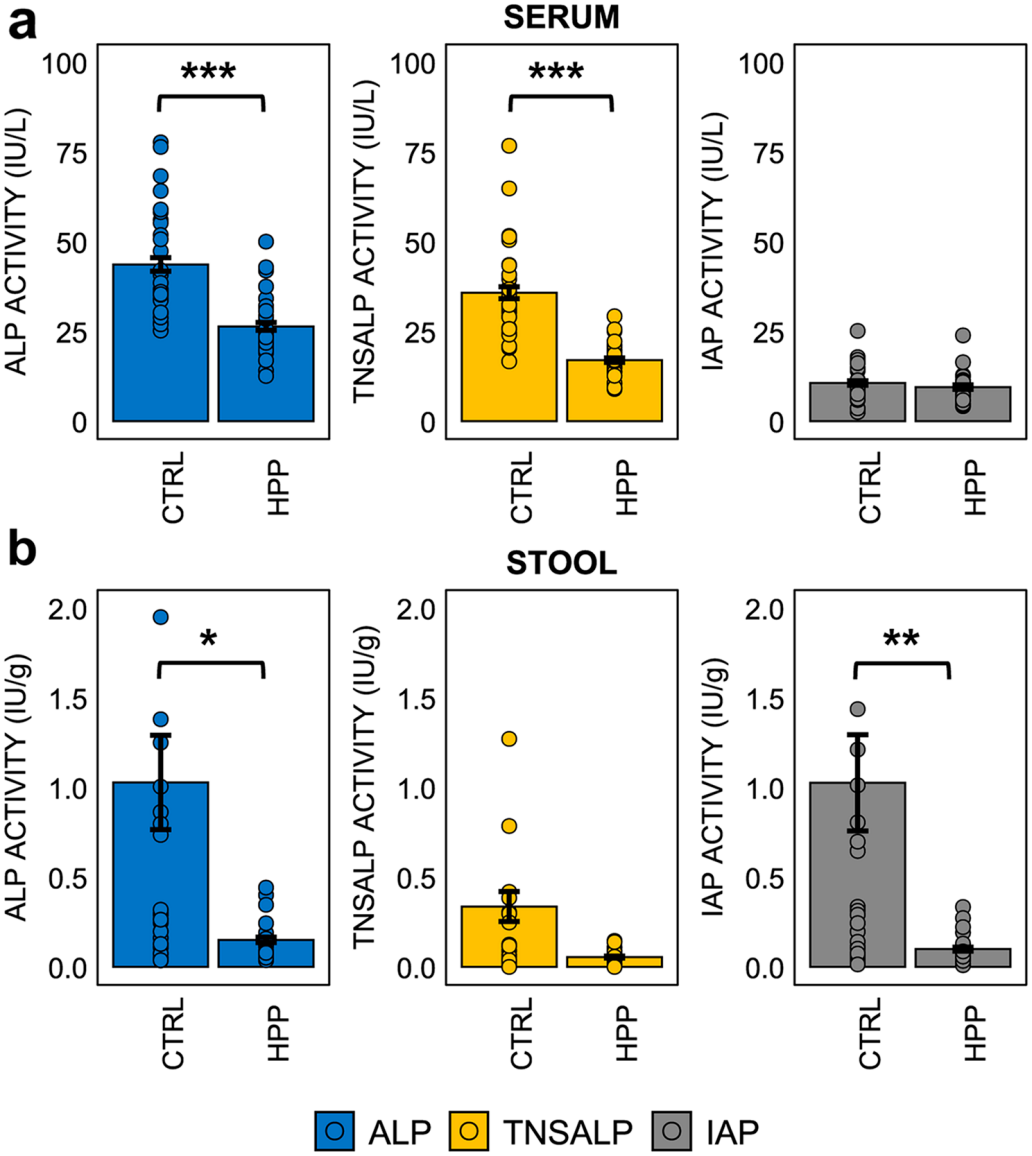
(**a**) Differences in total serum ALP, IAP and TNSALP activities between healthy controls (*n* = 30) and HPP patients (*n* = 30). (**b**) Differences in total ALP, IAP and TNSALP activities in stool between healthy controls (*n* = 29) and HPP patients (*n* = 25). A Mann-Whitney test was performed for two groups comparisons. *P*-value < 0.05 (*), < 0.01 (**), < 0.001 (***)

Serum total ALP activity was significantly lower in HPP patients than in controls, as expected (*p* < 0.001), TNSALP activity was significantly lower in all comparisons (*p* < 0.001). Regarding IAP, it showed a slight decrease in HPP patients compared to controls (*p* = 0.094) (Fig. 3a).

In the HPP group, total ALP activity in stools was significantly lower compared to the control group (*p* = 0.042). Similarly, IAP activity was significantly reduced in the HPP group compared to controls (*p* = 0.007). However, no significant differences were observed in TNSALP activity between the HPP and control groups (*p* = 0.1004) (Fig. 3b).

### Multivariate linear regression analysis of ALP isoenzymes in serum and feces

Multiple linear regression analysis revealed that both TNSALP and IAP activities are independent and significant predictors of total serum ALP levels. TNSALP showed the strongest contribution, indicating that both isoenzymes contribute positively and independently to serum ALP activity. The regression model demonstrated an excellent fit, explaining 90.3% of the variability in serum ALP levels, with no additional significant effects from confounding variables such as age or sex. These results are detailed in Table 1.

**Table 1:** Predictive variables of total ALP serum levels

	ALP (*R*^2^ = 0.9396)		
	β [95% CI]	*p*	VIF
Intercept	4.595[0.930–8.260]	0.015	
IAP	0.745[0.526–0.964]	< 0.001	1.281
TNSALP	0.876 [0.795–0.956]	< 0.001	1.301
AGE	-0.032[-0.919–3.113]	0.263	1.11
SEX	1.097[-0.089–0.025]	0.28	1.097

Multivariable linear regression of total serum ALP. ALP: total alkaline phosphatase; IAP: intestinal alkaline phosphatase; TNSALP: tissue non-specific alkaline phosphatase; CI: confidence interval; VIF: variance inflation factor

Furthermore, a strong positive association was identified between TNSALP and IAP activities in serum, suggesting a physiological interplay between both isoenzymes. Specifically, for every unit increase in TNSALP activity, serum IAP levels increased proportionally by an average of 0.164 units. However, this secondary model explained a more modest proportion of IAP variability (adjusted R² = 0.3633), implying that other regulatory mechanisms may also influence IAP levels (Table 2).

**Table 2:** Predictive variables of serum IAP levels

	IAP (*R*^2^ = 0.3633)		
	β [95% CI]	*p*	VIF
Intercept	4.286[1.463–7.108]	0.003	
TNSALP	0.164[0.108–0.221]	< 0.001	1.025
AGE	0.028[-0.0185–0.074]	0.232	1.085
SEX	-1.027[-2.643–0.589]	0.208	1.105

Multivariable linear regression of total serum ALP. IAP: intestinal alkaline phosphatase; TNSALP: tissue non-specific alkaline phosphatase; CI: confidence interval; VIF: variance inflation factor

For fecal samples, linear regression analyses using log10-transformed data showed that IAP activity was the principal determinant of total ALP levels. A highly significant association was observed, indicating that a 10-fold increase in IAP levels was associated with an approximate 8.3-fold increase in fecal ALP activity. This model exhibited an excellent fit (adjusted R² = 0.965), highlighting the dominant role of IAP in intestinal ALP activity. By contrast, no significant relationship was found between fecal TNSALP and total ALP (Table 3).

**Table 3:** Predictive variables of total stool ALP activity

	Log10(ALP) (*R*^2^ = 0.9695)		
	β [95% CI]	*p*	VIF
Intercept	0.011[-0.93–0.115]	0.834	
Log10 (IAP)	0.921[0.829–1.013]	< 0.001	3.186
Log10 (TNSALP)	-0.051[-0.120–0.018]	0.145	3.314
AGE	0[-0.002–0.001]	0.761	1.058
SEX	0.005[-0.064–0.074]	0.883	1.075

Multivariable linear regression of total serum ALP. ALP: total alkaline phosphatase; IAP: intestinal alkaline phosphatase; TNSALP: tissue non-specific alkaline phosphatase; CI: confidence interval; VIF: variance inflation factor

Notably, a robust and significant association was identified between fecal TNSALP and fecal IAP activities (B = 0.883; 95% CI: 0.747–1.019; *p* < 0.001), with each 10-fold increase in TNSALP corresponding to an average increase of 7.62-fold increase in IAP. This model explained 80.0% of the variability in fecal IAP levels (adjusted R² = 0.8002), supporting a physiologically relevant interaction between both isoenzymes at the intestinal level (Table 4).

**Table 4:** Predictive variables of stool IAP levels

	Log10(IAP) (*R*^2^ = 0.8002)		
	β [95% CI]	*p*	VIF
Intercept	0.315[0.019–0.611]	0.0375	
Log10(TNSALP)	0.883[0.747–1.020]	< 0.001	1.009
AGE	-0.003[-0.008–0.002]	0.242	1.011
SEX	0.112[-0.060–0.285]	0.196	1.011

Multivariable linear regression of total serum ALP. IAP: intestinal alkaline phosphatase; TNSALP: tissue non-specific alkaline phosphatase; CI: confidence interval; VIF: variance inflation factor

### Bone and inflammatory markers associated with the activity of ALP isoenzymes in serum and stools of HPP patients

HPP patients presented a distinct biochemical profile characterized by elevated PLP and iPTH levels and reduced 25(OH)D levels and BALP isoform. From an inflammatory standpoint, both IL-6 and fecal calprotectin were elevated beyond reference values. All measured variables are detailed in the Supplementary Material (Table S1).

To further investigate the relationship between ALP isoenzyme activity and clinical parameters, Spearman’s correlation analyses were conducted. These analyses included measurements of total ALP activity in serum and feces, as well as the specific isoenzymes TNSALP and IAP, in relation to markers of inflammation and bone metabolism.

**Fig. 4:**
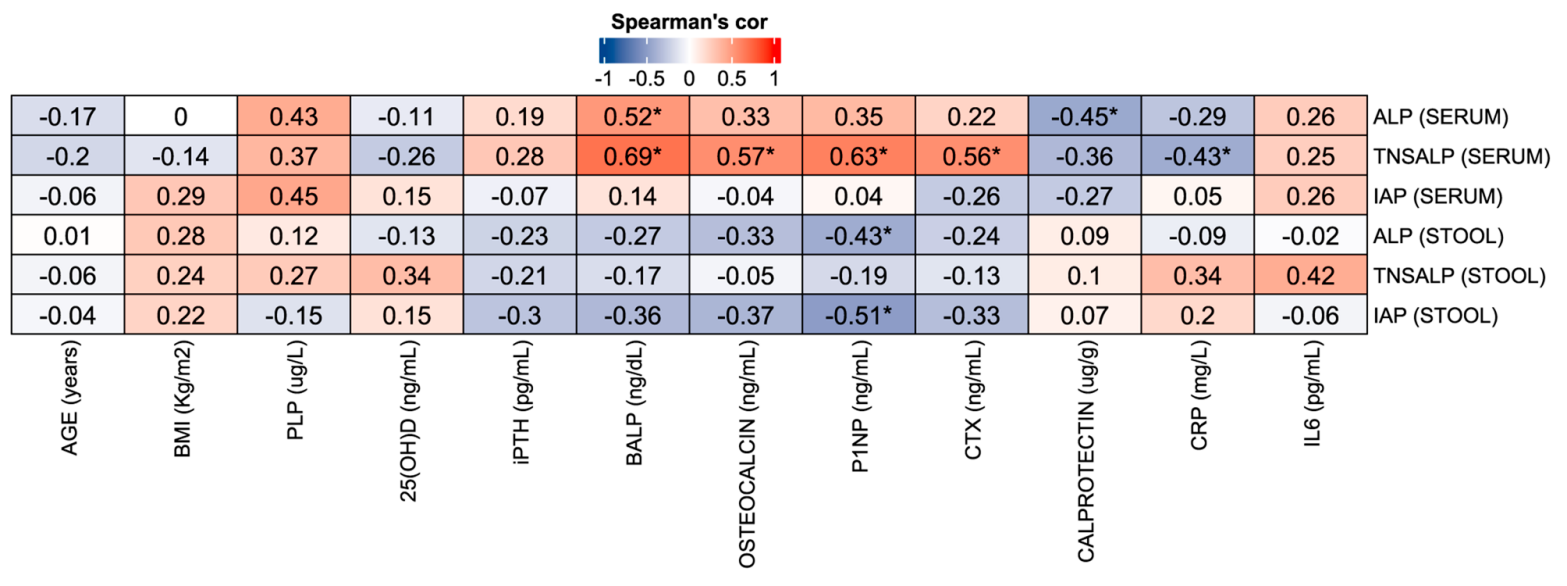
Spearman’s rho correlation between ALP activities with biochemical parameters in serum and stool. (* = *p* < 0.05). 25(OH)D: 25-hydroxyvitamin D, iPTH: intact parathyroid hormone, BALP: bone-specific alkaline phosphatase, P1NP: amino-terminal propeptide of procollagen type 1, CTX: Carboxy-terminal Telopeptide of Collagen Type I, CRP: C-reactive protein, IL6: Interleukin 6

As shown in Fig. 4, serum total ALP activity was positively correlated with fecal calprotectin and BALP levels. Serum TNSALP activity also demonstrated significant positive correlations with several bone turnover markers, including BALP, P1NP, and CTX, as well as with the inflammatory marker CRP.

By contrast, no significant associations were observed for serum IAP or fecal TNSALP activities with any of the bone or inflammatory markers analyzed. Interestingly, in fecal samples, both total ALP and IAP activities were inversely correlated with P1NP levels.

## Discussion

This study offers a detailed characterization of the ALP isoenzymes in serum and feces, providing new insights into HPP-related enzymatic profiles. Our findings confirm a marked reduction in serum TNSALP activity in HPP patients and identify concurrent alterations in IAP, indicating that the systemic effects of TNSALP deficiency extend beyond skeletal manifestations.

Under physiological conditions, TNSALP is the major ALP isoenzyme in serum [21]. IAP activity is produced by the pancreas, liver and kidney [22] beyond the intestine, but its contribution to serum ALP is minimal [23, 24]. By contrast, IAP constitutes the main source of ALP activity in feces [20, 25]. Any TNSALP activity detected in fecal samples could be attributable, at least in part, to bacterial ALP produced by members of the gut microbiota. Several Gram-negative bacteria, including Escherichia coli, Bacteroides spp., and some Bacillus strains, are known ALP producers [26–28]. Although bacterial ALP could contribute to ALP activity in stool, our data reinforce the role of IAP as the main contributing isoenzyme in stool under physiological conditions [29] and TNSALP represents a minimal contribution in this context.

Multiple linear regression analyses demonstrated that both serum TNSALP and IAP act as independent predictors of total ALP activity in serum. Additionally, TNSALP activity was found to predict serum IAP activity. However, model fitting suggests that other, yet unidentified, factors may substantially influence serum IAP regulation. In the fecal samples, IAP was the only isoenzyme significantly associated with total ALP activity, with an excellent model fit. Notably, multivariable regression revealed that fecal TNSALP activity was a predictor of fecal IAP levels independently of age and sex. These results reveal differential contributions and interrelationships of TNSALP and IAP in serum and fecal compartments, suggesting distinct but complementary physiological roles for each isoenzyme.

Some studies have reported that the increase in fecal IAP activity might reflect a compensatory or protective host response to modulate bacterial enzyme activity and maintain intestinal homeostasis under conditions of microbial imbalance or overgrowth [30]. In this context, increased IAP has been observed to promote the growth of intestinal bacteria in mice through nucleotide triphosphate dephosphorylation [29].

By contrast, our findings demonstrate a significant reduction in fecal IAP activity in HPP patients, suggesting an impairment of this protective intestinal mechanism. Beyond its potential contribution to intestinal dysbiosis, a marked decrease in IAP activity is biologically relevant, as it may trigger a cascade of downstream alterations that have not previously been considered in the context of HPP and are often interpreted as unrelated comorbidities. Reduced IAP activity has been associated with increased intestinal permeability [31], altered cholesterol metabolism [32], and a higher susceptibility to metabolic syndrome through mechanisms involving insulin resistance [7] and obesity [33] in murine models. In addition, low IAP activity predisposes to enhanced intestinal inflammation and has been linked to accelerated intestinal aging [31]. Notably, our results indicate that, rather than compensating for TNSALP deficiency in HPP patients, IAP activity appears to be influenced by TNSALP activity. This relationship suggests that reduced TNSALP may lead to a secondary decrease in IAP activity, thereby contributing to the systemic manifestations of the disease. This interpretation is consistent with the high prevalence of digestive and inflammatory alterations reported in patients with HPP [12, 19].

Agreeing, our results show that total serum ALP activity is related to fecal calprotectin, which is considered an intestinal inflammatory biomarker [34], whereas serum TNSALP activity is more related to bone metabolism. Although the relationship between serum TNSALP activity and fecal calprotectin did not reach statistical significance (probably due to the limited sample size of 30), the trend became significant when total serum ALP was analyzed. This suggests that TNSALP plays a predominant role in serum activity. Furthermore, TNSALP exhibited a significant inverse correlation with CRP, indicating that reduced TNSALP activity in pathological conditions may contribute to inflammation. Nevertheless, these correlation analyses should be interpreted with caution, as the observed correlation coefficients were in the low-to-moderate range (*r* < 0.5). Therefore, despite their statistical significance, the strength of these associations is moderate and may be overestimated in the context of a relatively small sample size, highlighting the need for validation in larger cohorts.

Notably, in fecal samples, both total ALP and IAP activities exhibited a significant inverse correlation with serum P1NP levels, a key marker of bone formation. Since IAP is the predominant ALP isoenzyme in the intestine, it is biologically plausible that the association observed between total fecal ALP and P1NP is mainly driven by IAP activity. Accordingly, the correlation observed for fecal IAP reached a moderate-to-strong magnitude (*r* > 0.5), whereas the association for total fecal ALP was weaker (*r* = 0.43), likely reflecting the contribution of non-IAP components to total ALP activity. Therefore, although both associations reached statistical significance, the lower correlation coefficient observed for total fecal ALP suggests a partial dilution of the relationship and indicates that this finding should be interpreted with caution. These findings suggest a potential link between intestinal enzymatic activity and systemic bone remodeling in patients with HPP. Although causality cannot be determined, the inverse relationship that was observed may be indicative of subclinical intestinal alterations that negatively influence bone formation. These alterations may be due to impaired nutrient absorption or low-grade intestinal inflammation. This hypothesis aligns with the concept of the gut–bone axis, which has been previously described in conditions such as inflammatory bowel disease [35] and metabolic syndrome [36], where chronic intestinal inflammation is associated with reduced bone formation. In these cases, chronic intestinal inflammation has been associated with reduced bone formation. In this context, IAP plays a central role in maintaining intestinal homeostasis, which is essential for adequate bone health. This is because efficient absorption of calcium, phosphate, and other nutrients critical for bone mineralization primarily occurs in the intestine [37]. Consequently, reduced IAP activity may contribute to an inflammatory intestinal environment, which can hinder nutrient uptake and potentially have a negative impact on bone formation and mineralization. Taken together, our results suggest that intestinal dysfunction may influence bone remodeling processes in HPP through alterations in IAP activity.

To date, no literature has described the molecular regulatory mechanisms linking these ALP isoenzymes, and this is the first study to investigate the impact of TNSALP deficiency on IAP activity. A hypothetical explanation is that reduced TNSALP activity may promote a chronic low-grade inflammatory state that secondarily suppresses IAP expression through NF-κB–mediated pathways. Activation of NF-κB increases the bioactive form of IL-1β [38] and activates TNF-α expression [39], both of which have been shown to downregulate IAP. In this context, NF-κB activation could arise from excessive stimulation of TLR4 by bacterial LPS [40] or from ATP binding to purinergic P2 × 7 [41] receptor (both LPS and extracellular ATP are known substrates of TNSALP [42]). Thus, impaired TNSALP activity may lead to the accumulation of these pro-inflammatory molecules, enhancing TLR4 and purinergic signaling and thereby triggering NF-κB activation. Consequently, the loss of TNSALP would not directly repress IAP but would create an inflammatory environment that, via NF-κB signaling, contributes to reduced IAP expression.

Some limitations of this study should be acknowledged to appropriately contextualize the findings. First, although the sample size is in line with other studies in rare diseases, a larger cohort would be desirable to strengthen the statistical robustness further and improve the generalizability of the results. In addition, the cross-sectional design limits the ability to draw temporal or causal conclusions regarding the relationship between TNSALP and IAP activities, highlighting the value of future longitudinal studies. Another aspect to consider is the lack of direct characterization of the microbiota. Given the known influence of the intestinal microbiota on intestinal alkaline phosphatase activity, future studies would benefit from the direct characterization of the intestinal microbiota to better understand its role in the regulation of intestinal alkaline phosphatase activity. This approach may also help to elucidate potential microbiota-mediated mechanisms linking hypophosphatasia to intestinal homeostasis and inflammation. Moreover, several regression models yielded very high adjusted R² values (> 0.9), which may partially reflect the intrinsic biochemical dependency between the analyzed isoenzymes, particularly in the context of the moderate sample sizes included in the study. As a formal cross-validation strategy was not implemented, the possibility of some degree of model overfitting cannot be completely ruled out. Therefore, these models should be interpreted primarily as descriptive of the observed biochemical relationships rather than as predictive tools. Future validation in independent and larger cohorts will be necessary to assess the robustness and predictive stability of these findings.

It is worth noting that, in the context of HPP, the decrease in TNSALP activity is not accompanied by a compensatory increase in IAP activity. On the contrary, IAP activity is also significantly reduced, particularly in the intestinal environment. These results contradict those of Parlato et al., who observed increased TNSALP expression in the mucosa of patients with IAP loss-of-function mutations [20]. This absence of compensatory response may play a role in the development of low-grade intestinal inflammation. Previous studies have indicated a link between reduced IAP activity and mucosal immune dysregulation [8, 43]. For instance, studies have shown that individuals with type 2 diabetes mellitus have reduced IAP levels [7, 25]. This condition is often linked to low-grade gut inflammation and dysbiosis [44]. Furthermore, genetic mutations affecting IAP expression have been involved in the pathogenesis of ulcerative colitis [20, 45], underscoring the enzyme’s pivotal role in preserving intestinal barrier integrity and immune tolerance. Research has shown that there is a link between reduced IAP activity and necrotizing enterocolitis and Crohn’s disease [46, 47]. Specifically, the decline in IAP activity corresponds with the severity of the disease and impaired intestinal defense mechanisms. Therefore, in HPP, the concurrent reduction in both TNSALP and IAP activity may exacerbate susceptibility to intestinal inflammation, further expanding the systemic implications of this enzymatic deficiency beyond bone metabolism.

## Conclusions

Our findings reveal a distinct enzymatic pattern in HPP, driven by profound TNSALP deficiency and accompanied by reductions in IAP activity, particularly at the intestinal environment. The decrease in the coordinated activity between these isoenzymes in HPP, together with associated systemic and intestinal alterations, underscores the importance of TNSALP not only in bone mineralization but also in broader physiological processes. These data do not support a compensatory relationship between TNSALP and IAP but instead suggest that the loss of TNSALP activity disrupts the equilibrium and functional interplay of ALP isoenzymes. Thus, rather than a compensatory response, the systemic impact of TNSALP deficiency also affects IAP activity, possibly through shared regulatory mechanisms favoring the development of an inflammatory environment in HPP patients.

## Supplementary Information

Below is the link to the electronic supplementary material.

Supplementary Material 1

Supplementary Material 2

## Data Availability

All data generated or analysed during this study are included in this published article (Supplementary Data).

## Abbreviations

ALP: Alkaline phosphatase
TNSALP: Tissue non-specific alkaline phosphatase
IAP: Intestinal alkaline phosphatase
PALP: Placental alkaline phosphatase
GCALP: Germ-cell alkaline phosphatase
HA: Hydroxyapatite
LPS: Lipopolysaccharide
HPP: Hypophosphatasia
CRP: C-reactive protein
IL6: Interleukin 6
BALP: Bone-specific alkaline phosphatase
CTX: Carboxy-terminal Telopeptide of Collagen Type I
P1NP: Amino-terminal propeptide of procollagen type I
iPTH: Intact parathyroid hormone
25(OH)D: 25-hydroxyvitamin D
PLP: Pyridoxal 5-phosphate
HPLC: High-pressure liquid chromatography
VIF: Variance inflation factor

## References

[1] Le Du M-H, Millan JL. Structural evidence of functional divergence in human alkaline phosphatases. J Biol Chem. 2002;277:49808–14. 10.1074/jbc.M207394200. 12372831 10.1074/jbc.M207394200

[2] Whyte MP. Hypophosphatasia - aetiology, nosology, pathogenesis, diagnosis and treatment. Nat Rev Endocrinol. 2016;12:233–46. 10.1038/nrendo.2016.14. 26893260 10.1038/nrendo.2016.14

[3] Li H, Zhao Y, Li W, Yang J, Wu H. Critical role of neutrophil alkaline phosphatase in the antimicrobial function of neutrophils. Life Sci. 2016;157:152–7. 10.1016/j.lfs.2016.06.005. 27287680 10.1016/j.lfs.2016.06.005

[4] Hernández-Chirlaque C, Gámez-Belmonte R, Ocón B, Martínez-Moya P, Wirtz S, Sánchez de Medina F, et al. Tissue Non-specific alkaline phosphatase expression is needed for the full stimulation of T cells and T Cell-Dependent colitis. J Crohns Colitis. 2017;11:857–70. 10.1093/ecco-jcc/jjw222. 28039309 10.1093/ecco-jcc/jjw222

[5] Shanmugham LN, Petrarca C, Castellani ML, Symeonidou I, Frydas S, Vecchiet J, et al. IL-1beta induces alkaline phosphatase in human phagocytes. Arch Med Res. 2007;38:39–44. 10.1016/j.arcmed.2006.05.016. 17174721 10.1016/j.arcmed.2006.05.016

[6] Millán JL, Whyte MP. Alkaline phosphatase and hypophosphatasia. Calcif Tissue Int. 2016;98:398–416. 10.1007/s00223-015-0079-1. 26590809 10.1007/s00223-015-0079-1 PMC4824800

[7] Gao C, Koko MYF, Ding M, Hong W, Li J, Dong N, et al. Intestinal alkaline phosphatase (IAP, IAP Enhancer) attenuates intestinal inflammation and alleviates insulin resistance. Front Immunol [Internet] Front. 2022. [cited 2025 Feb 12];13. 10.3389/fimmu.2022.927272 10.3389/fimmu.2022.927272 PMC9359302 35958560

[8] Estaki M, DeCoffe D, Gibson DL. Interplay between intestinal alkaline phosphatase, diet, gut microbes and immunity. World J Gastroenterol [Internet]. Baishideng Publishing Group Inc.; 2014 [cited 2025 Feb 12];20:15650–6. 10.3748/wjg.v20.i42.15650. 10.3748/wjg.v20.i42.15650 PMC4229529 25400448

[9] McConnell RE, Higginbotham JN, Shifrin DA Jr, Tabb DL, Coffey RJ, Tyska MJ. The enterocyte Microvillus is a vesicle-generating organelle. J Cell Biology [Internet]. 2009 [cited 2025 Feb 12];185:1285–98. 10.1083/jcb.200902147. 10.1083/jcb.200902147 PMC2712962 19564407

[10] Villa-Suárez JM, García-Fontana C, Andújar-Vera F, González-Salvatierra S, de Haro-Muñoz T, Contreras-Bolívar V, et al. Hypophosphatasia: A unique disorder of bone mineralization. Int J Mol Sci. 2021;22:4303. 10.3390/ijms22094303. 33919113 10.3390/ijms22094303 PMC8122659

[11] Mornet E, Taillandier A, Domingues C, Dufour A, Benaloun E, Lavaud N, et al. Hypophosphatasia: a genetic-based nosology and new insights in genotype-phenotype correlation. Eur J Hum Genet [Internet]. Nat Publishing Group; 2021. [cited 2023 Nov 1];29:289–99. 10.1038/s41431-020-00732-6. 10.1038/s41431-020-00732-6 PMC7868366 32973344

[12] González-Cejudo T, Villa-Suárez JM, Ferrer-Millán M, Andújar-Vera F, Contreras-Bolívar V, Andreo-López MC, et al. Mild hypophosphatasia May be twice as prevalent as previously estimated: an effective clinical algorithm to detect undiagnosed cases. Clin Chem Lab Med [Internet]. De Gruyter; 2023 [cited 2023 Oct 31]. 10.1515/cclm-2023-0427. 10.1515/cclm-2023-0427 37440753

[13] Uday S, Matsumura T, Saraff V, Saito S, Orimo H, Högler W. Tissue non-specific alkaline phosphatase activity and mineralization capacity of bi-allelic mutations from severe perinatal and asymptomatic hypophosphatasia phenotypes: results from an in vitro mutagenesis model. Bone [Internet]. 2019 [cited 2023 Oct 11];127:9–16. 10.1016/j.bone.2019.05.031. 31146036 10.1016/j.bone.2019.05.031

[14] Barvencik F, Beil FT, Gebauer M, Busse B, Koehne T, Seitz S, et al. Skeletal mineralization defects in adult hypophosphatasia–a clinical and histological analysis. Osteoporos Int. 2011;22:2667–75. 10.1007/s00198-011-1528-y. 21267545 10.1007/s00198-011-1528-y

[15] Martins L, Rodrigues TL, Ribeiro MM, Saito MT, Giorgetti APO, Casati MZ, et al. Novel ALPL genetic alteration associated with an odontohypophosphatasia phenotype. Bone [Internet]. 2013 [cited 2023 Oct 11];56:390–7. 10.1016/j.bone.2013.06.010. 23791648 10.1016/j.bone.2013.06.010 PMC3872001

[16] Dahir KM, Seefried L, Kishnani PS, Petryk A, Högler W, Linglart A, et al. Clinical profiles of treated and untreated adults with hypophosphatasia in the global HPP registry. Orphanet J Rare Dis [Internet]. 2022 [cited 2023 Oct 11];17:277. 10.1186/s13023-022-02393-8. 35854311 10.1186/s13023-022-02393-8 PMC9295501

[17] Cundy T, Michigami T, Tachikawa K, Dray M, Collins JF, Paschalis EP, et al. Reversible deterioration in hypophosphatasia caused by renal failure with bisphosphonate treatment. J Bone Min Res. 2015;30:1726–37. 10.1002/jbmr.2495. 10.1002/jbmr.2495 25736332

[18] Colazo JM, Hu JR, Dahir KM, Simmons JH. Neurological symptoms in hypophosphatasia. Osteoporos Int. 2019;30:469–80. 10.1007/s00198-018-4691-6. 30215116 10.1007/s00198-018-4691-6

[19] García-Fontana C, Villa-Suárez JM, Andújar-Vera F, González-Salvatierra S, Martínez-Navajas G, Real PJ, et al. Epidemiological, clinical and genetic study of hypophosphatasia in A Spanish population: identification of two novel mutations in the Alpl gene. Sci Rep. 2019;9:9569. 10.1038/s41598-019-46004-2. 31267001 10.1038/s41598-019-46004-2 PMC6606844

[20] Parlato M, Charbit-Henrion F, Pan J, Romano C, Duclaux-Loras R, Le Du M-H, et al. Human ALPI deficiency causes inflammatory bowel disease and highlights a key mechanism of gut homeostasis. EMBO Mol Med. 2018;10:e8483. 10.15252/emmm.201708483. 29567797 10.15252/emmm.201708483 PMC5887907

[21] Sato M, Saitoh I, Kiyokawa Y, Iwase Y, Kubota N, Ibano N et al. Tissue-Nonspecific alkaline Phosphatase, a possible mediator of cell maturation: towards a new Paradigm. cells [Internet]. 2021 [cited 2024 Sep 30];10:3338. 10.3390/cells10123338 10.3390/cells10123338 PMC8699127 34943845

[22] Domar U, Nilsson B, Baranov V, Gerdes U, Stigbrand T. Expression of intestinal alkaline phosphatase in human organs. Histochem [Internet]. 1992 [cited 2025 Feb 13];98:359–64. 10.1007/BF00271071 10.1007/BF00271071 1293076

[23] It-Koon T, Moss DW. The Estimation of intestinal alkaline phosphatase in human blood serum. Clin Chim Acta [Internet]. 1969 [cited 2025 Feb 28];25:117–25. 10.1016/0009-89816990236-8. 5797113 10.1016/0009-8981(69)90236-8

[24] Griffiths WC, Camara PD, Rosner M, Lev R, Brooks EM. Prevalence and properties of the intestinal alkaline phosphatase identified in serum by cellulose acetate electrophoresis. Clin Chem [Internet]. 1992 [cited 2025 Jul 1];38:507–11. 10.1093/clinchem/38.4.507. 1568315

[25] Malo MS. A High Level of Intestinal Alkaline Phosphatase Is Protective Against Type 2 Diabetes Mellitus Irrespective of Obesity. eBioMedicine [Internet]. Elsevier; 2015 [cited 2025 Feb 12];2:2016–23. 10.1016/j.ebiom.2015.11.027 10.1016/j.ebiom.2015.11.027 PMC4703762 26844282

[26] Derman AI, Beckwith J. Escherichia coli alkaline phosphatase localized to the cytoplasm slowly acquires enzymatic activity in cells whose growth has been suspended: a caution for gene fusion studies. J Bacteriol [Internet] Am Soc Microbiol. 1995 [cited 2025 Jun 14];177:3764–70. 10.1128/jb.177.13.3764-3770.1995. 10.1128/jb.177.13.3764-3770.1995 PMC177094 7601842

[27] Ichikawa T, Freese E. Alkaline phosphatase production of *Bacillus subtilis*. Biochim Et Biophys Acta (BBA) - Gen Subj [Internet]. 1974 [cited 2025 Jun 14];338:473–9. 10.1016/0304-41657490308-0.

[28] Yamashita Y, Toyoshima K, Yamazaki M, Hanada N, Takehara T. Purification and characterization of alkaline phosphatase of bacteroides gingivalis 381. Infection and immunity [Internet]. Am Soc Microbiol; 1990 [cited 2025 Jun 26];58:2882–7. 10.1128/iai.58.9.2882-2887.1990. 10.1128/iai.58.9.2882-2887.1990 PMC313582 2117573

[29] Malo MS, Moaven O, Muhammad N, Biswas B, Alam SN, Economopoulos KP, et al. Intestinal alkaline phosphatase promotes gut bacterial growth by reducing the concentration of luminal nucleotide triphosphates. Am J Physiol Gastrointest Liver Physiol [Internet]. Am Physiol Soc; 2014 [cited 2025 Feb 28];306:G826–38. 10.1152/ajpgi.00357.2013. 10.1152/ajpgi.00357.2013 PMC4024727 24722905

[30] Malo MS, Alam SN, Mostafa G, Zeller SJ, Johnson PV, Mohammad N, et al. Intestinal alkaline phosphatase preserves the normal homeostasis of gut microbiota. Gut [Internet] BMJ Publishing Group. 2010 [cited 2025 Jul 1];59:1476–84. 10.1136/gut.2010.211706. 10.1136/gut.2010.211706 20947883

[31] Kühn F, Adiliaghdam F, Cavallaro PM, Hamarneh SR, Tsurumi A, Hoda RS et al. Intestinal alkaline phosphatase targets the gut barrier to prevent aging. JCI insight [Internet]. American Society for Clinical Investigation; 2020 [cited 2025 Dec 10];5. 10.1172/jci.insight.134049. 10.1172/jci.insight.134049 PMC7213802 32213701

[32] Takeuchi A, Oda N, Takada K, Mori R, Aida T, Banno A, et al. Intestinal alkaline phosphatase is a receptor for cholesterol-lowering pentapeptide IIAEK and regulates cholesterol homeostasis in mice. Sci Rep [Internet] Nat Publishing Group. 2025 [cited 2025 Dec 10];15:20345. 10.1038/s41598-025-04722-w. 10.1038/s41598-025-04722-w PMC12217679 40594298

[33] Kaliannan K, Hamarneh SR, Economopoulos KP, Nasrin Alam S, Moaven O, Patel P, et al. Intestinal alkaline phosphatase prevents metabolic syndrome in mice. Proceedings of the National Academy of Sciences [Internet]. Proceedings of the National Academy of Sciences; 2013 [cited 2025 Dec 10];110:7003–8. 10.1073/pnas.1220180110. 10.1073/pnas.1220180110 PMC3637741 23569246

[34] Dajti E, Frazzoni L, Iascone V, Secco M, Vestito A, Fuccio L, et al. Systematic review with meta-analysis: diagnostic performance of faecal calprotectin in distinguishing inflammatory bowel disease from irritable bowel syndrome in adults. Alimentary Pharmacology & Therapeutics [Internet]. 2023 [cited 2025 Jun 26];58:1120–31. 10.1111/apt.17754. 10.1111/apt.17754 37823411

[35] Szafors P, Che H, Barnetche T, Morel J, Gaujoux-Viala C, Combe B, et al. Risk of fracture and low bone mineral density in adults with inflammatory bowel diseases. A systematic literature review with meta-analysis. Osteoporos Int [Internet]. 2018 [cited 2025 Jun 26];29:2389–97. 10.1007/s00198-018-4586-6. 29909470 10.1007/s00198-018-4586-6

[36] Lee C-Y, Chuang Y-S, Lee C-H, Wu M-T. Linking metabolic syndrome with low bone mass through insights from BMI and health behaviors. Sci Rep [Internet] Nat Publishing Group. 2023 [cited 2025 Jun 26];13:14393. 10.1038/s41598-023-41513-7. 10.1038/s41598-023-41513-7 PMC10474022 37658154

[37] Rizzoli R, Biver E, Brennan-Speranza TC. Nutritional intake and bone health. The Lancet Diabetes & Endocrinology [Internet]. Elsevier; 2021 [cited 2025 Jun 26];9:606–21. 10.1016/S2213-85872100119-4. 10.1016/S2213-8587(21)00119-4 34242583

[38] Guo H, Callaway JB, Ting JP-Y. Inflammasomes: mechanism of action, role in disease, and therapeutics. Nat Med [Internet] Nat Publishing Group. 2015 [cited 2025 Dec 10];21:677–87. 10.1038/nm.3893. 10.1038/nm.3893 PMC4519035 26121197

[39] Arkoudi K, Yuan Y, Cumine AP, Dyer C, Busch-Nentwich E, Bravo I, et al. An NF-kB/TNF-alpha signalling feedback loop acts to coordinate tissue regeneration and macrophage behaviour in zebrafish. Npj Regen med [Internet]. Nature Publishing Group; 2025 [cited 2025 Dec 10];10:27. 10.1038/s41536-025-00414-1. 10.1038/s41536-025-00414-1 PMC12134371 40461478

[40] Faure E, Equils O, Sieling PA, Thomas L, Zhang FX, Kirschning CJ, et al. Bacterial lipopolysaccharide activates NF-κB through Toll-like receptor 4 (TLR-4) in cultured human dermal endothelial cells: Differential expression of TLR-4 and TLR-2 in endothelial cells*. J Biol Chem [Internet]. 2000 [cited 2025 Dec 10];275:11058–63. 10.1074/jbc.275.15.11058. 10753909 10.1074/jbc.275.15.11058

[41] Liu J, Liu S, Hu S, Lu J, Wu C, Hu D, et al. ATP ion channel P2X purinergic receptors in inflammation response. Biomed Pharmacotherapy [Internet]. 2023 [cited 2025 Dec 10];158:114205. 10.1016/j.biopha.2022.114205. 10.1016/j.biopha.2022.114205 36916431

[42] Peters E, Geraci S, Heemskerk S, Wilmer MJ, Bilos A, Kraenzlin B, et al. Alkaline phosphatase protects against renal inflammation through dephosphorylation of lipopolysaccharide and adenosine triphosphate. Br J Pharmacol [Internet]. 2015 [cited 2024 Jan 22];172:4932–45. 10.1111/bph.13261. 26222228 10.1111/bph.13261 PMC4621995

[43] Lallès J-P. Intestinal alkaline phosphatase: novel functions and protective effects. Nutr Reviews [Internet]. 2014 [cited 2025 Jun 26];72:82–94. 10.1111/nure.12082. 10.1111/nure.12082 24506153

[44] Al Bataineh MT, Künstner A, Dash NR, Alsafar HS, Ragab M, Schmelter F, et al. Uncovering the relationship between gut microbial dysbiosis, metabolomics, and dietary intake in type 2 diabetes mellitus and in healthy volunteers: a multi-omics analysis. Sci Rep [Internet] Nat Publishing Group. 2023 [cited 2025 Jun 26];13:17943. 10.1038/s41598-023-45066-7. 10.1038/s41598-023-45066-7 PMC10589304 37863978

[45] Tuin A, Poelstra K, de Jager-Krikken A, Bok L, Raaben W, Velders MP, et al. Role of alkaline phosphatase in colitis in man and rats. Gut [Internet] BMJ Publishing Group; 2009 [cited 2025 Jun 26];58:379–87. 10.1136/gut.2007.128868. 10.1136/gut.2007.128868 18852260

[46] Heath M, Buckley R, Gerber Z, Davis P, Linneman L, Gong Q et al. Association of intestinal alkaline phosphatase with necrotizing Enterocolitis among premature infants. JAMA Network Open [Internet]. 2019 [cited 2025 Jun 26];2:e1914996. 10.1001/jamanetworkopen.2019.14996. 10.1001/jamanetworkopen.2019.14996 PMC6902776 31702803

[47] Park S-Y, Kim J-Y, Lee S-M, Chung JO, Seo J-H, Kim S, et al. Lower expression of endogenous intestinal alkaline phosphatase May predict worse prognosis in patients with crohn’s disease. BMC Gastroenterol [Internet]. 2018 [cited 2025 Jun 26];18:188. 10.1186/s12876-018-0904-x. 30558547 10.1186/s12876-018-0904-x PMC6296121

