# Supplementary material for: Tissue nonspecific and intestinal alkaline phosphatase crosstalk: a missing link in hypophosphatasia pathophysiology?
Source: J Transl Med. 2026 Feb 8;24:354. doi: 10.1186/s12967-026-07791-1 (PMC12977700; doi:10.1186/s12967-026-07791-1)

Table S1. Biochemical parameters of HPP patients.

|  | Mean | SEM | Reference Range |
| --- | --- | --- | --- |
| AGE (years) | 48.20 | 2.63 | > 18 years |
| BMI (kg/m2) | **27.37*** | 0.76 | 21-24 kg/m2 |
| PLP (ug/L) | **78.56*** | 8.56 | 15-50 ug/L |
| 25(OH)D (ng/mL) | **27.49*** | 1.27 | 30-100 ng/mL |
| iPTH (pg/mL) | **66.91*** | 7.68 | 15-65 pg/mL |
| BALP (ng/dL) 5.7 | **4.98*** | 0.36 | 5.7-20.9 ng/mL |
| OSTEOCALCIN (ng/mL) | 23.17 | 1.43 | 11-43 ng/mL |
| P1NP (ng/mL) | 59.08 | 5.78 | 16-96 ng/mL |
| CTX (ng/mL) | 0.55 | 0.06 | 0.112-1.008 ng/mL |
| CALPROTECTIN (ug/g) | **359.69*** | 104.34 | < 50 ug/g |
| CRP (mg/L) | 4.48 | 0.79 | < 5 mg/L |
| IL6 (pg/mL) | **6.08*** | 1.34 | < 5.9 pg/mL |

BMI: Body mass index, PLP: Pyridoxal 5-phosphate, 25(OH)D: 25-hydroxyvitamin D, iPTH: intact parathyroid hormone, BALP: bone-specific alkaline phosphatase, P1NP: amino-terminal propeptide of procollagen type 1, CTX: carboxy-terminal telopeptide of collagen type I, CRP: C-reactive protein, IL6: interleukin 6 (*) Parameters outside of the reference value.

Figure S1: Plots illustrating the homoscedasticity and normality of residuals for each of the regression models in serum and stool samples


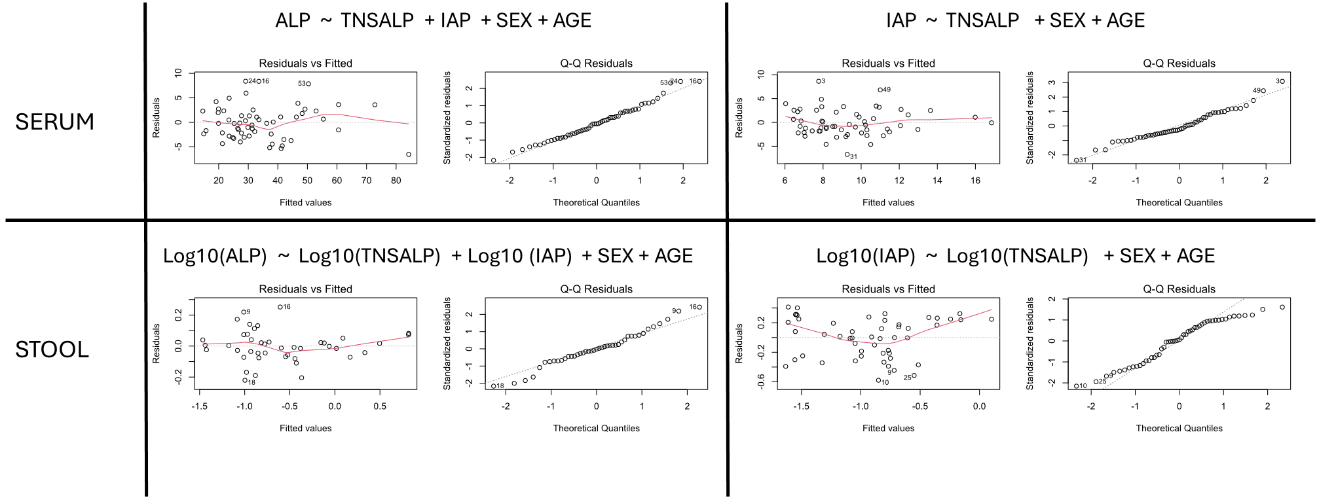

Supplement: Supplementary file 2 — Supplementary Material 2 [file 12967_2026_7791_MOESM2_ESM.docx]
